# Validation of Health Belief Model and Theory of Planned Behaviour Constructs for Predicting Malaria Vaccine Acceptance in Ghana: A Partial Least Squares Structural Equation Modelling Approach

**DOI:** 10.1111/tmi.70193

**Published:** 2026-06-30

**Authors:** Michael Sarfo, Michael Snowden, Jamie P. Halsall

**Affiliations:** ^1^ School of Human and Health Sciences University of Huddersfield Huddersfield UK

**Keywords:** Ghana, health belief model, malaria vaccine, PLS‐SEM, theory of planned behaviour, vaccine hesitancy

## Abstract

**Aim:**

This study examines the predictive power of constructs from the health belief model (HBM) and theory of planned behaviour (TPB) in explaining malaria vaccine acceptance in Ghana in the post‐COVID‐19 era, while also assessing the reliability of these constructs within this context.

**Methods:**

A cross‐sectional survey of 622 adults was conducted in two Ghanaian municipalities representing high‐ and low‐malaria endemicity. Constructs from HBM and TPB, alongside contextual factors such as religiosity and traditional medicine use, were measured. Partial Least Squares Structural Equation Modelling (PLS‐SEM) was used to assess construct validity and estimate their predictive relationships with vaccine intention.

**Results:**

All constructs demonstrated strong reliability (Cronbach's *α* = 0.894–0.985) and convergent validity (AVE = 0.797–0.971). The model demonstrated strong predictive power, explaining 67.6% of the variance in vaccine intentions (*R* ^2^ = 0.676). Attitudes (*β* = 0.578, *p* < 0.001) and perceived benefits (*β* = 0.531, *p* < 0.001) were the strongest predictors of acceptance. Perceived behavioural control had a marginal effect (*β* = 0.124, *p* = 0.064). Perceived disease vulnerability negatively predicted intention (*β* = −0.330, *p* = 0.006), while perceived severity and subjective norms were non‐significant.

**Conclusion:**

HBM and TPB constructs are reliable and valid in predicting malaria vaccine acceptance in Ghana. Interventions should focus on strengthening positive attitudes and highlighting the benefits of vaccination, while addressing misconceptions among those who perceive themselves as most vulnerable. This study provides novel evidence on malaria vaccine acceptance in a post‐pandemic context and underscores the utility of combining HBM and TPB for predicting preventive health behaviours in sub‐Saharan Africa.

## 1 Introduction

Malaria is a parasitic infection caused by various strains of Plasmodium species, such as Falciparum, Vivax, Ovale and Malariae, transmitted through the bite of an infected female anopheles mosquito [1]. According to the World Malaria report 2024, in 2023, there were ~263 million malaria cases globally and a staggering 597,000 malaria‐related deaths across 83 countries [2]. The report further estimates a disproportionately high burden of malaria cases in the African region, accounting for about 246 million (94%) cases and 569,000 (95%) deaths, with a heavy toll on children under 5 years, who account for 80% of all malaria‐related deaths in the region [2]. Alarmingly, the 2025 World Health Organisation malaria report estimated an additional 9 million malaria cases worldwide, reflecting a 3% increase compared with previous estimates and highlighting the persistent challenge of malaria control efforts [3]. In Ghana, a previous study conducted by Iddrisu and Moyer [4] asserts that over 10.4 million outpatient (OPD) visits per year are attributed to malaria infection, with children under 5 years accounting for about 4.2% of all cases.

In view of the morbidity and mortality associated with malaria, particularly among vulnerable populations such as children, the World Health Organisation recommends two malaria vaccines to reduce the frequency and severity of malaria infections [5]. These vaccines are the RTS,S/ASO1 (Mosquirix) and R21/Matrix‐M. After a successful piloting test in 42 districts in seven regions, Ghana became the first country to approve the malaria vaccine for use in children [6, 7, 8].

Anecdotal studies posit that the implementation of the malaria vaccine is feasible to deliver via the routine immunisation programme and offers a positive public health impact when merged with other preventive interventions. For instance, findings from a multi‐country study in Ghana, Kenya and Malawi highlighted that a significant vaccine uptake and a subsequent reduction in complicated malaria and hospitalisations in implementation areas versus non‐vaccinated areas, with no records of safety concerns [9]. The study further estimates the coverage of Dose 1 (76%) and Dose 3 (66%) among eligible children in Ghana, which is higher than other similar expanded programme on immunisation (EPI) antigens in comparable settings, reducing the incidence of malaria significantly [9]. Other previous studies [7, 10] have noted that community engagement, health system strengthening, clear communication strategies and equity in vaccine access are key elements that reinforce intentions to accept or reject the malaria vaccine among caregivers.

However, prior to the COVID‐19 pandemic, countries such as Ghana had adopted pragmatic strategies to eliminate malaria through programmes such as the setting up of the National Malaria Elimination Programme (NMEP), which derives its objectives from the global agenda of eliminating malaria by 2030, as enshrined in the sustainable development Goal 3 (SDG3) [11]. Despite this, previous studies have demonstrated a significant drop‐out rate of malaria testing, malaria treatment and health service utilisation during the pandemic. For example, Hakizimana et al. [12] assert a decrease in malaria testing of an estimated 4.32 per 1000 patients before the pandemic compared to an increase of 2.38 per 1000 during the pandemic in high endemic regions in Rwanda. Other previous studies [13, 14, 15] have posited that the advent of COVID‐19 has heightened vaccine hesitancy among diverse socio‐economic backgrounds. To further support these earlier studies, Ghazy et al. [16] noted in their study on monkeypox vaccine acceptance among Ghanaians that about 53.9% of the study population did not intend to accept the vaccine if it became available. The study further illuminates that gender, previous coronavirus vaccine refusal, and level of education are the key reasons for the refusal to accept the monkeypox vaccine. Against the backdrop of the complexities of vaccine hesitancy, particularly in the post‐pandemic period, increased vaccine drop‐out rates and zero‐ dose cases, and the re‐emergence of infectious diseases, this study seeks to contribute to and inform the debate on increasing vaccine hesitancy and provide new knowledge and insights into the drivers of malaria vaccine uptake. This will be achieved by axiomatizing two theories: the theory of planned behaviour (TPB) and the health belief model (HBM) to predict intentions to accept the malaria vaccine from contrasting malaria endemicity. The combined use of the HBM and the TPB provides a robust theoretical framework for understanding vaccine hesitancy in Ghana, as it bridges individual health‐related risk–benefit assessments with broader social and volitional determinants of behaviour. While HBM constructs such as perceived benefits emphasise the utilitarian value of vaccination in preventing infection [17], the TPB attitude construct captures individuals' internal psychological evaluations of the act of vaccination itself [18]. This integrated framework is particularly relevant in the post‐pandemic era, where vaccine decisions are increasingly shaped by complex psychological dispositions, trust dynamics and behavioural intentions rather than the fear of disease. To the best of our knowledge, only one study [19] has applied the TPB to examine malaria vaccine uptake in Ghana, while no known study has applied either the HBM independently or an integrated HBM–TPB framework within the context of malaria vaccine acceptance in Ghana. Consequently, this study positions itself as a pioneering effort by being the first to formally validate and apply an integrated HBM–TPB construct to predict malaria vaccine acceptance in the Ghanaian context.

## 2 Method

### 2.1 Study Design and Setting

This cross‐sectional study employed a multistage sampling approach anchored in Probability Proportional to Size (PPS) principles to ensure demographic and geographic representativeness [20]. In the first stage, the Ashanti and Oti regions were purposively selected to represent distinct strata of low and high malaria endemicity, respectively, as defined by the National Malaria Elimination Programme. In the second stage, one municipality was randomly selected from each region (Kwadaso and Jasikan). Simple random sampling was applied at this stage to ensure that each eligible municipality within the selected regions had an equal probability of selection, followed by the identification of 14 clusters (8 in Kwadaso and 6 in Jasikan) based on high‐traffic public areas and residential density.

The PPS approach was instrumental in ensuring that the urban and more populous Kwadaso Municipality contributed a larger proportional share of participants than the rural Jasikan municipality. This weighting reflects the reality that urban centres often act as primary hubs for healthcare dissemination and vaccine rollout. Within these clusters, a systematic selection procedure was employed where every third adult who passed a designated point or entered a household was approached, reducing selection bias compared to pure convenience sampling. Participants were recruited from multiple community settings, including marketplaces, bus stations, community centres and individual households.

### 2.2 Sample Size Determination

The required sample size for this study was calculated using the single population proportion formula:

$$n=Z^{2}\;x\;p\;x\;\left(1-p\right)/E^{2}$$

where *n* = required sample size; *Z* = standard normal deviation corresponding to a 95% confidence level (1.96); *p* = estimated proportion of participants willing to accept a malaria vaccine; *E* = margin of error, set at 0.05.

The relationship between the projected proportion of interest (*p*) and the required sample size (*n*) indicates that the maximum sample size is reached when *p* = 0.5. At this point, the required sample size is ~384 participants. To account for potential non‐responses and attrition, the sample size was increased by ~20%, resulting in a target sample size of roughly 460. The final adjusted target was rounded to 500 participants to ensure sufficient power.

### 2.3 Participants and Data Collection

Participants were included in the study if they were 18 years and above and less than 60 years, lived in either Kwadaso or Jasikan Municipality for at least 6 months and verbally consented to take part in the study. However, participants were ineligible for the study if they had not lived in the study settings for at least 6 months, showed signs of intoxication or mental incapacity based on the objective assessment by research assistants or did not meet the age range required for the study. Data was collected by the researcher and five trained research assistants using paper‐based questionnaires.

### 2.4 Study Questionnaire and Mitigation of Survey Bias

To address common challenges in self‐reported data, such as social desirability and acquiescence bias, several psychometric safeguards were implemented. The study utilised paper‐based questionnaires that collected no identifiable personal information, maintaining strict participant anonymity to encourage honest reporting on the sensitive topic of vaccine hesitancy. To mitigate acquiescence bias, the survey instrument incorporated a diverse range of items, specifically employing both standard and reverse‐coded Likert scales for key constructs such as perceived benefits and vulnerability, while those related to attitudes, subjective norms, perceived behavioural control, religiosity and traditional medicine use followed a standard ‘strongly agree’ to ‘strongly disagree’ format. This design acted as a cognitive safeguard, enabling participants to engage more deeply with the content and reducing the risk of automatic ‘strongly agree’ response patterns [21]. Furthermore, trained research assistants emphasised confidentiality during recruitment in diverse public settings and households to foster an environment of trust.

### 2.5 Dependent Variable

The dependent variable is the degree of respondents' intentions to accept the malaria vaccine. In the survey, the respondents were asked to rate their health status on a 4‐likert scale with the following response: 1 = Yes, definitely; 2 = Yes probably; 3 = No probably not and 4 = No definitely not. These were recorded in SPSS during the analysis to 4 = Yes, definitely; 3 = Yes probably; 2 = No probably not and 1 = No definitely not.

### 2.6 Independent Variables

The study adopted three constructs and three items each from the TPB and HBM and other sociodemographic variables. The constructs adopted for the purposes of this study are attitudes, subjective norms, perceived behavioural control, perceived disease severity, perceived benefits and perceived vulnerability using a five Likert scale. Sociodemographic variables measured in this study include age (1 = 18–29, 2 = 30–44, 3 = 45–59), gender (1 = Male, 2 = Female), educational level (1 = No formal education, 2 = Primary, 3 = Junior high, 4 = Secondary, 4 = Tertiary), Residence (1 = Kwadaso, 2 = Jasikan), Past covid‐19 vaccination history (1 = Yes, 2 = No), Heard of the malaria vaccine (1 = Yes, 2 = No, 3 = Not sure).

### 2.7 Data Analysis

Data were analysed using Partial Least Squares Structural Equation Modelling (PLS‐SEM) with SmartPLS (v.4.0). PLS‐SEM was chosen over covariance‐based SEM because it is particularly suitable for exploratory research, models with both reflective constructs and complex structural relationships, and studies with relatively smaller sample sizes and non‐normally distributed data. In this study, PLS‐SEM was employed to validate constructs derived from the HBM and the TPB, and to examine their predictive power for malaria vaccine acceptance. Unlike traditional regression approaches, which primarily examine direct relationships between observed variables, PLS‐SEM enables the simultaneous assessment of both measurement reliability and structural relationships among multiple latent constructs. Compared to covariance‐based SEM (CB‐SEM), PLS‐SEM is particularly advantageous for exploratory and prediction‐oriented research where the primary objective is to maximise the explained variance (*R* ^2^) of dependent constructs [22]. The technique is also robust in handling non‐normally distributed data and complex behavioural models involving multiple reflective constructs, conditions commonly encountered in public health survey research. Furthermore, the use of behavioural intention as a proxy for actual vaccine uptake is theoretically and empirically supported by the TPB, which identifies intention as the most immediate antecedent of health‐related behaviour [18]. This alignment justifies the use of intention as a valid outcome measure for assessing the potential success of the malaria vaccine rollout in Ghana.

INT=β1ATT+β2PB2+β3PBC+β4PDS+β5PDV+B6SBN+ς

where INT = intentions to accept malaria vaccine; ATT = attitudes towards the malaria vaccine; PB = perceived benefits; PBC = perceived behavioural control; PDS = perceived disease severity; PDV = perceived disease vulnerability; *ζ* = error term; *β* = path coefficient.

#### 2.7.1 Measurement Model

The first step involved evaluating the measurement model to ensure that the constructs exhibited adequate reliability and validity. Reliability was assessed using Cronbach's alpha and Composite Reliability (CR), with thresholds of ≥ 0.70 considered acceptable. Convergent validity was examined through the Average Variance Extracted (AVE), where values above 0.50 indicate that the construct explains more than half of the variance of its indicators. Discriminant validity was assessed using the Fornell–Larcker criterion and inspection of cross‐loadings, confirming that each construct captured a distinct dimension of vaccine‐related beliefs.

All HBM and TPB constructs (attitudes, perceived benefits, perceived behavioural control, perceived disease severity, perceived disease vulnerability and subjective norms) exceeded the recommended thresholds (Cronbach's alpha range = 0.894–0.985; CR > 0.90; AVE = 0.797–0.971), thereby confirming strong reliability and convergent validity. Discriminant validity was also established, as the square root of each construct's AVE was greater than its correlations with other constructs.

#### 2.7.2 Structural Model Assessment

Multicollinearity was assessed using Variance Inflation Factor (VIF), with all values below the conservative threshold of 5. Path coefficients were estimated using bootstrapping with 5000 resamples, which generated standard errors, *t*‐statistics and bias‐corrected confidence intervals. The model explained 67.6% of the variance in malaria vaccine intention (*R* ^2^ = 0.676; adjusted *R* ^2^ = 0.673), indicating strong explanatory power.

## 3 Results

Table 1 presents malaria vaccine acceptance across sociodemographic and contextual characteristics. Acceptance (‘Yes definitely’) was highest among younger respondents aged 18–29 (82.3%) and lowest among those aged 30–44 (58.5%) and 45–59 (63.1%). Males (74.6%) were more likely than females (67.8%) to report definite acceptance. Education strongly influenced intention: respondents with secondary (83.0%) and tertiary education (76.4%) showed the highest definite acceptance, while those with no formal education (18.9%) and junior high education (4.0%) reported the lowest.

**TABLE 1:** Malaria vaccine acceptance by sociodemographic and contextual characteristics (*n* = 622).

Variable	Yes definitely	Yes probably	No probably	No definitely not
Age group				
18–29	247 (82.3%)	44 (14.7%)	6 (2.0%)	3 (1.0%)
30–44	117 (58.5%)	46 (23.0%)	25 (12.5%)	12 (6.0%)
45–59	77 (63.1%)	18 (14.8%)	8 (6.6%)	19 (15.6%)
Gender				
Male	214 (74.6%)	37 (12.9%)	16 (5.6%)	20 (7.0%)
Female	227 (67.8%)	71 (21.2%)	23 (6.9%)	14 (4.2%)
Education level				
No formal education	17 (18.9%)	20 (22.2%)	23 (25.6%)	30 (33.3%)
Primary	9 (27.3%)	8 (24.2%)	7 (21.2%)	9 (27.3%)
Junior High	6 (4.0%)	3 (2.0%)	31 (20.7%)	110 (73.3%)
Secondary	146 (83.0%)	26 (14.8%)	3 (1.7%)	1 (0.6%)
Tertiary	146 (76.4%)	39 (20.4%)	5 (2.6%)	1 (0.5%)
Residence				
Kwadaso Municipal	306 (84.3%)	47 (12.9%)	7 (1.9%)	3 (0.8%)
Jasikan Municipal	135 (52.1%)	61 (23.6%)	32 (12.4%)	31 (12.0%)
Heard of malaria vaccine				
Yes	324 (90.3%)	31 (8.6%)	3 (0.8%)	1 (0.3%)
No	117 (44.5%)	77 (29.3%)	36 (13.7%)	33 (12.5%)
COVID‐19 vaccine acceptance				
Yes	379 (83.5%)	60 (13.2%)	7 (1.5%)	8 (1.8%)
No	62 (36.9%)	48 (28.6%)	32 (19.0%)	26 (15.5%)

*Note:* Values are presented as frequency (*n*) and percentage (%). Percentages are calculated within each category of the explanatory variable. ‘Yes definitely’ represents the strongest intention to accept the malaria vaccine, while ‘No definitely not’ represents the strongest intention to reject the vaccine. *Interpretation*: Higher percentages in the ‘Yes definitely’ category indicate stronger willingness to accept the malaria vaccine. Vaccine acceptance was generally higher among younger adults, respondents with secondary or tertiary education, residents of Kwadaso Municipality, individuals who had previously heard about the malaria vaccine and those who had accepted the COVID‐19 vaccine.

Abbreviation: COVID‐19, Coronavirus Disease 2019.

Regional variation was evident, with 84.3% of respondents in Kwadaso Municipality indicating definite acceptance compared to 52.1% in Jasikan. Prior awareness of the malaria vaccine was strongly associated with acceptance, as 90.3% of those who had heard of it indicated definite acceptance compared to 44.5% of those who had not. Similarly, COVID‐19 vaccine history predicted malaria vaccine acceptance: 83.5% of respondents who had received COVID‐19 vaccination reported definite acceptance of the malaria vaccine, compared to only 36.9% among those who had not.

Table 2 presents the reliability and validity statistics for the constructs of the HBM and TPB. All constructs demonstrated strong internal consistency, with Cronbach's alpha values ranging from 0.894 (perceived disease vulnerability) to 0.985 (perceived benefits), exceeding the recommended 0.70 threshold. Similarly, both composite reliability measures (ρa and ρc) were above the 0.70 cut‐off, indicating robust construct reliability.

**TABLE 2:** Reliability and validity of HBM and TPB.

Construct	Cronbach's alpha	Composite reliability (ρa)	Composite reliability (ρc)	Average variance extracted (AVE)
Attitude (ATT)	0.971	0.971	0.981	0.945
Perceived benefits (PB)	0.985	0.985	0.990	0.971
Perceived Behavioural Control (PBC)	0.960	0.963	0.974	0.927
Perceived Disease Severity (PDS)	0.983	0.993	0.989	0.967
Perceived Disease Vulnerability (PDV)	0.894	1.184	0.922	0.797
Subjective Norms (SBN)	0.962	0.962	0.975	0.929

*Note:* Cronbach's Alpha assesses internal consistency reliability, with values ≥ 0.70 considered acceptable. Composite reliability (ρa and ρc) evaluates construct reliability, with values ≥ 0.70 indicating satisfactory reliability. Average variance extracted (AVE) assesses convergent validity, with values ≥ 0.50 indicating adequate construct validity.*Interpretation*: As seen in this table, all constructs exceeded recommended thresholds for reliability and convergent validity, indicating that the measurement model was psychometrically robust and suitable for assessing malaria vaccine acceptance intentions in the Ghanaian context.

Abbreviations: ρa, Dijkstra–Henseler's rho; ρc, composite reliability; ATT, attitude towards malaria vaccination; AVE, average variance extracted; PB, perceived benefits; PBC, perceived behavioural control; PDS, perceived disease severity; PDV, perceived disease vulnerability; SBN, subjective norms.

Convergent validity was supported as all average variance extracted (AVE) values were well above the 0.50 benchmark, ranging from 0.797 (perceived disease vulnerability) to 0.971 (perceived benefits). Collectively, these results confirm that the measurement model demonstrates satisfactory reliability and convergent validity across all HBM and TPB constructs. It is worth noting that the unusually high ρa value for perceived disease vulnerability (1.184) may suggest item redundancy or collinearity among indicators. While this does not undermine the construct's reliability, it highlights the need for cautious interpretation and potential refinement of measurement items in future studies.

As indicated in Table 3, the validated constructs from HBM and TPB were then tested for their structural relationships with intention to accept the malaria vaccine (INT). Bootstrapping with 5000 resamples was used to assess the significance of path coefficients. The model demonstrated strong explanatory power with an *R* ^2^ of 0.676 (adjusted *R* ^2^ = 0.673), indicating that psychosocial constructs accounted for nearly 68% of the variance in vaccine intentions. Significant positive predictors of vaccine intention were attitude (*β* = 0.578, *p* < 0.001) and perceived benefits (*β* = 0.531, *p* < 0.001). Perceived behavioural control showed a positive but marginal effect (*β* = 0.124, *p* = 0.064). In contrast, perceived disease severity (*β* = −0.074, *p* = 0.179) and subjective norms (*β* = 0.023, *p* = 0.715) were not statistically significant. Surprisingly, perceived disease vulnerability exerted a significant negative effect on intentions (*β* = −0.330, *p* = 0.006), suggesting that individuals perceiving themselves as highly vulnerable to malaria were paradoxically less likely to accept the vaccine.

**TABLE 3:** Structural model path coefficients for predicting Malaria vaccine intention.

Path	*β*	Sample mean (M)	Standard deviation (SD)	*t*	*p*
ATT → INT	0.578	0.580	0.055	10.501	< 0.001
PB → INT	0.531	0.534	0.107	4.941	< 0.001
PBC → INT	0.124	0.125	0.067	1.851	0.064
PDS → INT	−0.074	−0.076	0.055	1.344	0.179
PDV → INT	−0.330	−0.332	0.121	2.733	0.006
SBN → INT	0.023	0.020	0.062	0.365	0.715

*Note:* Path coefficients (*β*) represent the strength and direction of relationships between latent constructs and malaria vaccine intention. Positive *β* values indicate an increase in intention, whereas negative *β* values indicate a decrease in intention. Statistical significance was determined using bootstrapping with 5000 resamples. *Interpretation*: Attitude and perceived benefits were the strongest positive predictors of malaria vaccine acceptance. Perceived behavioural control demonstrated a weaker positive association, while perceived disease vulnerability showed a significant negative relationship with vaccine intention. Perceived disease severity and subjective norms were not statistically significant predictors.

Abbreviations: *β*, standardised path coefficient; ATT, attitude; INT, intention to accept malaria vaccine; *p*, probability value; PB, perceived benefits; PBC, perceived behavioural control; PDS, perceived disease severity; PDV, perceived disease vulnerability; SBN, subjective norms; SD, standard deviation.

### 3.1 Model Explanatory Power

The model explained nearly 68% of the variance in malaria vaccine acceptance intentions (*R* ^2^ = 0.676), indicating a substantial explanatory strength. The final PLS‐SEM structural model for predicting malaria vaccine acceptance intention is presented in Figure 1.

**FIGURE 1:**
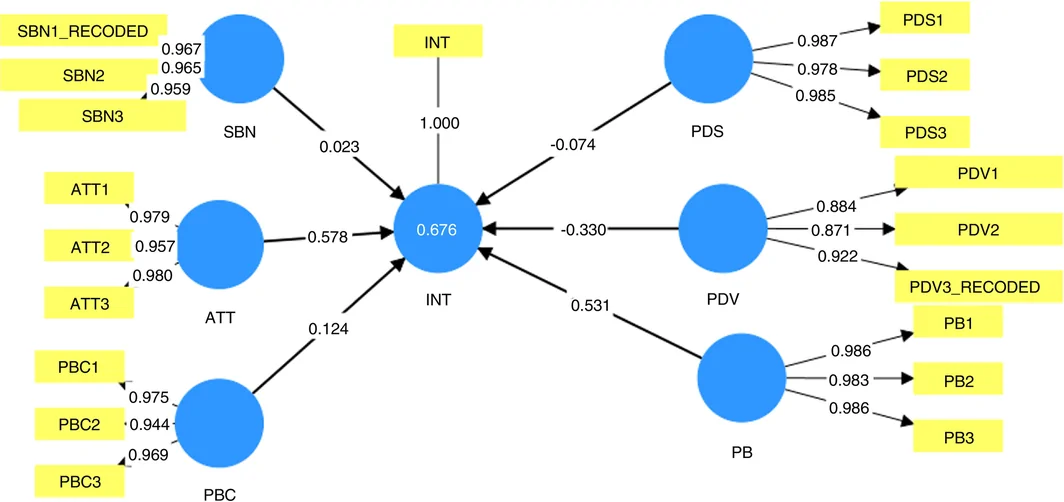
PLS SEM structural model for predicting malaria vaccine acceptance intention. The blue circles represent latent constructs included in the health belief model (HBM) and theory of planned behaviour (TPB). Yellow rectangles represent observed indicators (questionnaire items) used to measure each construct. These correspond to the key survey questions administered to participants and serve as the observable measures underlying the latent theoretical constructs. Numbers on the arrows linking indicators to constructs represent factor loadings, while numbers on arrows connecting constructs represent standardised path coefficients (*β*). The value displayed within the central endogenous construct (INT) represents the coefficient of determination (*R* ^2^). *Colour key*: Blue circles: Latent theoretical constructs. Yellow rectangles: Measured survey indicators/items. Black arrows: Structural and measurement relationships between constructs and indicators. *Interpretation*: The model explained 67.6% of the variance in malaria vaccine intention (*R* ^2^ = 0.676). Attitude and perceived benefits demonstrated the strongest positive effects on vaccine acceptance intentions, whereas perceived disease vulnerability exerted a significant negative effect. The high factor loadings indicate strong relationships between the observed items and their respective latent constructs.

Abbreviations: ATT, attitude; INT, intention to accept malaria vaccine; PB, perceived benefits; PBC, perceived behavioural control; PDS, perceived disease severity; PDV, perceived disease vulnerability; *R* ^2^, coefficient of determination; SBN, subjective norms; *β*, standardised path coefficient.

## 4 Discussion

### 4.1 Validation of HBM and TPB Constructs

This study first assessed the psychometric properties of constructs adapted from the HBM and the TPB. The results of the measurement model demonstrated that all constructs met or exceeded conventional thresholds for reliability and validity, with Cronbach's alpha values ranging from 0.894 to 0.985, composite reliability above 0.90, and average variance extracted (AVE) values ranging from 0.797 to 0.971. These findings confirm the robustness of the adopted measures in capturing key psychosocial dimensions of vaccine decision‐making.

The validation results are consistent with prior research across a range of health behaviours. For example, McEachan et al. [23] in a meta‐analysis of TPB applications found that attitude, perceived behavioural control and subjective norms consistently demonstrated high internal reliability across health contexts. Similarly, Jones et al. [24] found that the HBM confirmed that perceived benefits and perceived susceptibility constructs are generally reliable indicators in explaining preventive health behaviour. Our findings extend this evidence by demonstrating that these constructs are also reliable and valid in the Ghanaian context, where few validation studies have been conducted.

Furthermore, the high AVE values observed indicate that the constructs capture substantial variance in their indicators, supporting convergent validity. This aligns with past validation studies that have applied the HBM and TPB in diverse cultural settings, including studies on cervical cancer screening, nurses' health behaviour and HIV prevention [25, 26, 27]. Importantly, establishing discriminant validity indicates that each construct is conceptually distinct, reinforcing the theoretical soundness of combining HBM and TPB constructs within a single predictive framework.

Collectively, these findings make a methodological contribution by confirming that psychosocial constructs from the HBM and TPB retain strong psychometric properties in sub‐Saharan Africa. This strengthens the case for their continued use in explaining preventive health behaviours in culturally diverse populations.

### 4.2 Structural Model Results

After establishing construct validity, the study examined the predictive power of the HBM and TPB constructs for malaria vaccine acceptance. The structural model accounted for nearly 68% of the variance in predicting intentions to accept the malaria vaccine, which is considered substantial in behavioural science [22]. Attitudes towards vaccination and perceived benefits emerged as the strongest positive predictors of malaria vaccine acceptance. This mirrors findings from vaccine acceptance studies across settings, where favourable attitudes and recognition of health benefits consistently drive uptake [28, 29, 30]. The robustness of these predictors highlights the central role of shaping positive beliefs in vaccination campaigns.

Perceived behavioural control showed a weaker but positive effect, consistent with TPB assumptions [18]. Although marginally significant, this suggests that individuals who perceive fewer barriers to vaccine access are more likely to intend to accept it. For example, this relatively modest effect may reflect the influence of contextual and logistical constraints, including distance to health facilities, transportation challenges, waiting times and uncertainty regarding vaccine availability. In settings where access to healthcare services is uneven, individuals may express positive attitudes towards vaccination while still perceiving limited control over their ability to obtain the vaccine [19, 31]. The marginal significance of PBC may also indicate that, although access‐related concerns are relevant, they are secondary to stronger motivational factors such as attitudes and perceived benefits. Nevertheless, from a public health perspective, even modest access barriers can substantially affect uptake at the population level.

Prior studies on HPV and COVID‐19 vaccines similarly highlight that logistical barriers, costs and perceived ease of access significantly influence uptake decisions [32, 33]. This emphasises the need for malaria vaccination strategies that ensure affordability, accessibility and convenience.

Contrary to expectations, perceived disease vulnerability demonstrated a significant negative relationship with vaccine intentions. While the HBM predicts that higher perceived susceptibility motivates protective behaviour [34, 35], our finding suggests a paradox. One plausible interpretation is that individuals who perceive themselves as highly vulnerable may doubt the vaccine's effectiveness or prefer alternative coping strategies such as traditional medicine or prayer. This finding resonates with studies in other African contexts, where high perceived risk sometimes coexists with vaccine hesitancy due to distrust or competing explanatory models of illness [36, 37]. Again, in Ghana, concerns for vaccine safety and the growing level of mistrust in Western medicine continue to perpetuate. For example, Sarfo et al. [38] note that parental rejection of the HPV vaccine for young girls was deeply rooted in issues centring around lack of trust in vaccines and other misinformation such as the tendency of the vaccine to cause infertility in young children. Taking all these into consideration, individuals, even though they may be susceptible to the disease, would still not accept the vaccine if all these factors exist.

Conversely, perceived disease severity and subjective norms did not significantly predict vaccine intentions. These null findings suggest that while malaria is widely recognised as a serious disease, its severity alone does not motivate vaccine acceptance if individuals believe they already have other effective preventive options. In highly endemic settings such as Ghana, malaria may also be socially and epidemiologically normalised, such that perceptions of severity are relatively uniform across individuals and therefore less likely to differentiate behavioural intentions. As a result, perceived severity may function more as a background health belief than as an active driver of vaccination decisions. This finding is complemented by prior studies conducted in Egypt and Debrebrhan [39, 40]. Similarly, the lack of influence of subjective norms may reflect the high social normalisation of malaria in Ghana, limiting the impact of peer or community approval on individual vaccine decisions.

In such contexts, vaccination decisions may be shaped more by individuals' personal evaluations of the vaccine's effectiveness, perceived benefits and practical value than by social expectations or external pressure. This could be due to malaria being a familiar and recurrent condition in Ghana, making decisions about its prevention more individualised and experience‐driven rather than socially negotiated. Consequently, individuals may rely more heavily on their own assessment of whether the vaccine offers meaningful protection beyond existing preventive measures, rather than on the opinions or approval of peers, family members or the wider community. This contrasts with findings in contexts where novel vaccines such as COVID‐19 were highly socially contested [41].

Interestingly, the observed variation in malaria vaccine acceptance across sociodemographic and contextual factors also highlights the importance of situating vaccine decision‐making within broader structural and experiential contexts. In particular, the substantial difference between Kwadaso and Jasikan municipalities likely reflects underlying disparities in healthcare infrastructure, accessibility and exposure to health information. Kwadaso, being more urbanised, may benefit from stronger health system capacity and greater engagement with public health messaging, whereas more rural settings, such as Jasikan, may face logistical constraints and rely more heavily on local belief systems, which can shape perceptions of biomedical interventions like the malaria vaccination. This interpretation is consistent with evidence illustrating that health system capacity, access and community trust shape malaria vaccine uptake in Ghana [6, 10].

In addition, the strong association between prior COVID‐19 vaccination and malaria vaccine acceptance suggests a potential behavioural spillover effect, whereby previous vaccination experiences influence subsequent health decisions. Individuals who accepted the COVID‐19 vaccine may have developed greater trust in health systems, increased confidence in vaccine safety and familiarity with vaccination processes, thereby reinforcing their willingness to accept the malaria vaccine. Conversely, those who declined COVID‐19 vaccination may carry forward concerns related to safety, mistrust or misinformation, which could negatively influence acceptance. This aligns with broader evidence demonstrating that vaccine attitudes and trust established during the COVID‐19 pandemic continue to shape subsequent vaccination behaviours [14, 15, 16]. For instance, in a study published a few months before the pandemic was declared over, it found that participants who had refused the COVID‐19 vaccine had a higher likelihood of refusing the monkeypox vaccine if they were to be made available [16]. Together, these findings emphasise that malaria vaccine uptake is not only driven by individual beliefs but is also shaped by contextual inequalities and prior experiences with vaccination, highlighting the need for tailored, context‐sensitive public health strategies.

### 4.3 Strengths and Limitations

This study has several notable strengths. It represents the first empirical investigation in Ghana to validate and integrate constructs from both the HBM and the TPB for predicting malaria vaccine acceptance, thereby contributing new theoretical and methodological insights to the field of vaccine hesitancy research in sub‐Saharan Africa. The psychometric assessment was rigorous, employing PLS‐SEM, and the results demonstrated consistently strong reliability and validity across constructs. The relatively large sample of 622 participants, drawn from municipalities representing both high‐ and low‐endemicity regions, provides a solid basis for generalisation within the Ghanaian context.

Despite these strengths, certain limitations must be acknowledged. The cross‐sectional design restricts the ability to make causal inferences, as intentions may not always translate into actual vaccine behaviour. Reliance on self‐reported responses raises the possibility of social desirability or recall bias, even though the survey design attempted to minimise this risk through the use of reversed and standard Likert items. The focus on two municipalities, while capturing variation in malaria endemicity, may not fully reflect the diversity of the Ghanaian population, limiting the generalisability of the findings to other regions. Additionally, the unusually high reliability observed for some constructs, particularly perceived vulnerability, suggests potential redundancy in the measurement items and indicates the need to further refine these scales in future studies. Finally, the study did not test the moderating effects of sociodemographic characteristics such as gender, education or age, which might further enrich understanding of the heterogeneity in vaccine acceptance.

## 5 Conclusion

This study validated constructs from the HBM and the TPB and tested their predictive power for malaria vaccine acceptance in Ghana. Findings demonstrated that attitudes and perceived benefits were the strongest positive predictors, while perceived vulnerability unexpectedly reduced intentions. Perceived disease severity and subjective norms were not significant, underscoring the complexity of vaccine decision‐making in malaria‐endemic contexts. By integrating HBM and TPB, this study confirms the robustness of these psychosocial models in sub‐Saharan Africa and highlights their explanatory value for malaria vaccine acceptance in a post‐pandemic era. Practically, the findings suggest that communication strategies should emphasise the tangible benefits of vaccination and foster positive attitudes, while addressing misconceptions that may arise among those who feel most vulnerable. This evidence provides policymakers with an empirically grounded framework for strengthening malaria vaccine uptake, thereby supporting national and global malaria elimination goals. To translate these findings into practice, future malaria vaccination campaigns should prioritise communication strategies that emphasise the tangible benefits of vaccination, including protection against malaria infection, reduced disease burden and improved child and community health outcomes. Given the strong influence of attitudes on vaccine intentions, interventions should also focus on building confidence in vaccine safety and effectiveness through trusted healthcare providers, community engagement and culturally sensitive messaging. In addition, targeted efforts to address misinformation and improve accessibility may be particularly important in populations with high perceived vulnerability but lower vaccine acceptance. Together, these strategies may strengthen public confidence and support the successful implementation of malaria vaccination programmes in Ghana.

## Funding

The authors have nothing to report.

## Ethics Statement

The study adhered to all ethical guidelines for research involving human participants. Ethical approval was obtained from the University of Huddersfield's School Research Ethics and Integrity Committee (SREIC/2025/038) and from the Committee on Human Research, Publication and Ethics at Kwame Nkrumah University of Science and Technology, Ghana (254221411930). Only participants who consented to participate in the study were included in the data collection.

## Consent

The authors have nothing to report.

## Conflicts of Interest

The authors declare no conflicts of interest.

## Data Availability

The data that support the findings of this study are available from the corresponding author upon reasonable request.
